# Analysis of flavor formation and metabolite changes during production of Double-Layer Steamed Milk Custard made from buffalo milk

**DOI:** 10.1371/journal.pone.0331277

**Published:** 2025-09-08

**Authors:** Hong Lan, Xier Luo, Dingyun Xu, Kuiqing Cui, Ling Li, Chuan Li, Hao Qi, Qingyou Liu

**Affiliations:** 1 Guangdong Provincial Key Laboratory of Animal Molecular Design and Precise Breeding, School of Animal Science and Technology, Foshan University, Foshan, China; 2 Guangxi Buffalo Research Institute, Chinese Academy of Agricultural Sciences, Nanning, China; Yantai Institute of Technology, CHINA

## Abstract

Double-Layer Steamed Milk Custard (DLSMC) is a famous traditional Chinese dessert. This study aimed to analyze the flavor and the changes in metabolites during different stages of DLSMC preparation, including raw buffalo milk, thermo-processing, first and second-layer milk skin formation. Electronic nose and electronic tongue were employed to preliminarily assess the overall flavor characteristics between these stages. The results indicated that DLSMC demonstrated increased sweetness, saltiness, and umami compared to raw buffalo milk, along with heightened levels of nitrogen oxides, methane, alcohols, aldehydes, ketones, and sulfur-containing compounds. Thus, the thermo-processing and second-layer milk skin formation were pinpointed as critical for flavor alterations. Additionally, headspace-gas chromatography-ion mobility spectrometry (HS-GC-IMS) detected a total of 46 volatile organic compounds (VOCs), with 8 compounds identified as key flavor components. Untargeted metabolomics revealed 98 differential metabolites, including amino acids, lipids, and nucleotides, that were significantly linked to changes in key flavor compounds. Indeed, the fluctuations in the levels of amino acids, lipids, and nucleotides play a crucial role in influencing flavor changes during the production of DLSMC made from buffalo milk.

## Introduction

In southern China, buffalo farming has a rich history and offers significant economic benefits through the production of milk, meat, leather, and draft power [1]. As a significant component of global milk resources, buffalo milk has higher levels of fat than other milk [2]. Previous research indicates that buffalo milk is a rich source of polyunsaturated fatty acids and amino acids, both are beneficial for human lipid metabolism [3,4]. Buffalo milk, with its higher fat and protein content, is more suitable for the production of high-fat dairy products compared to other types of milk [5]. Buffalo milk mozzarella cheese was rich in various volatile flavor substances, including hexanal, heptanal, ethyl caproate, (E)-2-nonenal, furfural, and ethyl butyrate. Compared to mozzarella prepared from holstein milk, mozzarella prepared from buffalo milk exhibits a more complex aroma profile [6]. Double-Layer Steamed Milk Custard (DLSMC) is renowned as a “Famous Chinese Snack” in Guangdong province, China. It represents a key part of Chinese traditional dairy products, reflecting the essence of classic dairy culture. The technique of making DLSMC was included in the seventh batch of provincial intangible cultural heritage representative items in Guangdong province in 2018. DLSMC, made from buffalo milk, eggs, and sugar, is characterized by the formation of two layers on the surface during production. Raw buffalo milk is boiled and cooled to form the first layer of milk skin, which is then pierced to pour out the remaining milk. After adding eggs and sugar, the mixture is stirred and steamed, resulting in a second layer of milk skin. The method primarily involves heating, cooling, and stirring. The changes caused by heat treatment—such as microbial killing, whey protein and casein cross-linking, fat oxidative degradation, Maillard reaction, and thermal denaturation—can alter the flavor components and metabolites in milk [7–10].

A variety of analysis techniques have been widely used to detect volatile organic compounds (VOCs) responsible for the volatile flavor in milk and dairy products [11–14]. Electronic nose and electronic tongue are electronic instruments that mimic human sensory responses and provide objective analysis for flavor detection [15]. During the thermal treatment of milk, metabolites such as lipids and proteins can be easily oxidized to VOCs including aldehydes, alcohols and ketones [16]. Compared to gas chromatography (GC) and gas chromatography-mass spectrometry (GC-MS), headspace-gas chromatography-ion mobility spectrometry (HS-GC-IMS) provides several advantages, including with no preprocessing, rapid response times, high sensitivity and convenient operation [17]. Consequently, electronic nose, electronic tongue, and HS-GC-IMS were utilized to comprehensively analyze flavor formation in this study.

In recent years, non-targeted metabolomics has become a powerful tool for exploring metabolic changes under various conditions, particularly in complex samples [18,19]. Non-targeted metabolomics detects differential metabolites under different heat treatment conditions and establishes predictive models to effectively distinguish between pasteurized milk and overheated milk [20]. Additionally, it can be used to identify metabolites in buffalo milk across different varieties [21]. Dairy samples contain numerous small molecule metabolites with a wide range of dynamic changes in concentration and polarity. Therefore, the high sensitivity and resolution of mass spectrometry-based metabolomics make it particularly suitable for analyzing the metabolites in buffalo milk and dairy products. UHPLC-Orbitrap Exploris 240 MS shows great potential in analyzing the composition of buffalo milk.

This research was designed to investigate the variations in flavor and metabolites of DLSMC and analyzed correlation among them during the production process. In our study, the flavor and taste profiles at different production stages were analyzed using electronic nose and electronic tongue. The VOCs in the production process of DLSMC were determined by HS-GC-IMS. Non-targeted metabolomics technology using UHPLC-Orbitrap Exploris 240 MS was utilized to determine the alterations in metabolites during production. This study also explored the relationship between VOCs and metabolic compounds in DLSMC. This research proposes a novel perspective for investigating VOCs in DLSMC, providing a scientific basis for understanding the unique flavor and nutritional characteristics of DLSMC. Additionally, it establishes a theoretical framework for enhancing the processing methodology of DLSMC, as well as quality control throughout the processing.

## Materials and methods

### Preparation method for Double-Layer Steamed Milk Custard

Buffalo milk samples were collected from the buffalo breeding farm at the Buffalo Research Institute, Chinese Academy of Agricultural Sciences in Nanning, Guangxi, China. The method was derived from the traditional Double-Layer Steamed Milk Custard technique documented in Foshan Intangible Cultural Heritage Database (https://foshanfy.mgdatatech.com/feiyi/project/show/id/38.html). Raw milk is boiled for 10 minutes and cooled at 25°C for 30 minutes, during which a first layer of milk skin forms. A small hole is then pierced in the skin, allowing the buffalo milk to be poured out. The first layer of milk skin sticks to the bottom of the bowl. After adding eggs and sugar in the standardized ratio of raw buffalo milk: egg: sugar = 10:1:1 (w/w/w), the mixture is steamed in a pot for 20 minutes, leading to the formation of a second milk skin. Samples were collected at four key stages of the production process: raw buffalo milk (group A), thermo-processing (100°C for 10 minutes, group B), first-layer of milk skin formation (25°C for 30 minutes, group C), and second-layer of milk skin formation (100°C 20 minutes, group D). Three samples were collected at each stage.

### Detection of flavor by electronic nose

The AIRSENSE PEN 3 portable electronic nose (Germany) contains 10 sensors, including W1C, W5S, W3C, W6S, W5C, W1S, WIW, W2S, W2W, W3S. Ten sensors detect different types of VOCs, as shown in the S1 Table [10]. Weigh 5 g ± 0.1 g of the sample and equilibrate it at 40°C for 20 minutes before testing. The instrument parameters were configured as follows: flush time of 60 seconds, zero-point trim time of 5 seconds, measurement time of 180 seconds, and gas flow rate set to 300 mL/min. Every sample underwent once tests. Three relatively stable data were selected for further analysis using the Winmuster software (version 1.6.2.22; Airsense Analytics GmbH).

### Detection of taste by electronic tongue

The electronic tongue detection was conducted using the Taste-Sensing System SA 402B (Intelligent Sensor Technology Co., Ltd., Atsugi, Japan). The electronic tongue is equipped with six sensors: CT0 for saltiness, CA0 for sourness, AAE for umami and aftertaste-U, AE1 for astringency and aftertaste-A, C00 for bitterness and aftertaste-B, and GL1 for sweetness. Following dilution with water at a 1:1 ratio and centrifugation at 4500 rpm for 20 minutes, both the upper fat layer and lower solids were removed. A 30 mL ± 0.5 mL sample was placed in a sample test cup. All samples were tested four times. The first test data was discarded, and the data from the last three tests were used for analysis.

### Detection of volatile organic compounds by HS-GC-IMS

VOCs were analyzed by HS-GC-IMS (FlavourSpec®-G.A.S. Dortmund Company, Germany) according to Zhao's study [13]. A 2 g ± 0.05 g sample was placed in a 20 mL headspace vial, incubated at 80°C, and shaken at 500 rpm for 20 minutes. A 500 μL headspace sample was injected at an injection needle temperature of 85°C. The GC was fitted with a chromatographic column MXT-WAX (polar, 30 m × 0.53 mm × 1.0 μm; RESTEK, USA) and operated at 60°C for separation. Nitrogen (purity ≥99.999%) was used as the carrier gas, with an initial flow rate of 2 mL/min. The GC was operated under the following conditions: the flow rate was maintained at 2 mL/min for 2 minutes, from 2 mL/min to 10 mL/min within 8 min, from 10 mL/min to 100 mL/min within 10 min, from 100 mL/min to 150 mL/min within 10 min. The drift gas flow rate was set at 150 mL/min, while the column temperature was maintained at 60°C. A mixture of ketones, including 2-butanone, 2-pentanone, 2-hexanone, 2-heptanone, 2-octanone, and 2-nonanone (C4-C9), was used to formulate the column-modified curve for calculating the retention index (RI). LAV software (v2.2.1) was utilized for analyzing the HS-GC-IMS data, while GC × IMS Library Search (v1.0.3) was employed for qualitative analysis within the GC-IMS library. VOCs were identified by comparing retention indices (RI) and drift times (DT) with reference data from the GC-IMS spectrum library.

### Metabonomics analysis

Weigh 200 ± 5 mg of the sample precisely, and add 800 µL of 80% aqueous methanol solution (methanol: water = 4:1 (v:v), HPLC grade) containing the internal standard solution L-2-chlorophenylalanine (0.02 mg/mL, ≥ 98%). The tissue sample is ground for 6 minutes at −10°C and 50 Hz using a cryogenic grinder, followed by ultrasonic extraction for 30 minutes at 5°C and 40 kHz. After that, the sample is placed at −20°C for 30 minutes and centrifuged at 13,000 g and 4°C for 15 minutes. The supernatant is carefully collected for further analysis. A portion of the supernatant from each sample is combined to create a quality control (QC) sample. Analysis is performed on a UHPLC-Exploris 240 system (Thermo Fisher Scientific Inc., Waltham, MA, USA). Separation of 3 µL samples is achieved using an ACQUITY UPLC HSS T3 column (100 mm × 2.1 mm, 1.8 µm, Waters, Milford, USA), followed by mass spectrometric detection. The column is maintained at 40°C. Mass spectra are acquired in both positive and negative ion modes, covering a mass range of 70–1050 m/z. The sheath gas flow is set at 60 psi, auxiliary gas at 20 psi, and the auxiliary gas temperature at 350°C. Ion spray voltages are set to 3400 V for positive mode and −3000 V for negative mode, while the ion transfer tube is heated to 350°C. The primary and secondary mass spectrometric resolutions are set at 60,000 and 15,000 respectively. Each sample were detected once in the data dependent acquisition (DDA) mode.

### Data analysis

The raw data was processed using the Progenesis QI metabolomics software (Waters Corporation, Milford, USA), which performed baseline filtering, peak identification, integration, retention time correction, and peak alignment. The MS and MS/MS mass spectrometry information were then compared with public databases such as Human Metabolome Database (HMDB) (http://www.hmdb.ca/), Metlin (https://metlin.scripps.edu/), as well as a self-built database. Following the database search, the resulting matrix data was uploaded to a cloud platform (https://cloud.majorbio.com) for further analysis. During data preprocessing, the 80% rule was applied to exclude missing values. To mitigate errors from sample preparation and instrument variation, the response intensity of key spectral peaks was normalized using the sum normalization method. Variables with a relative standard deviation (RSD) greater than 30% in QC samples were removed, and log10 transformation was performed on the data.

Multivariate statistical methods, including unsupervised principal component analysis (PCA) and orthogonal partial least squares-discriminant analysis (OPLS-DA), were conducted using the SIMCA-P 14.1. Differential metabolites were selected based on the variable importance in projection (VIP) value from the OPLS-DA analysis and p-values obtained from the Student's t-test. Metabolites with VIP > 1 and p < 0.05 were classified as differential. Metabolic pathways for the differential metabolites were identified through annotation using the Kyoto Encyclopedia of Genes and Genomes (KEGG) database (https://www.kegg.jp/kegg/pathway.html).

## Results and discussion

### Electronic nose analysis

**Fig 1:**
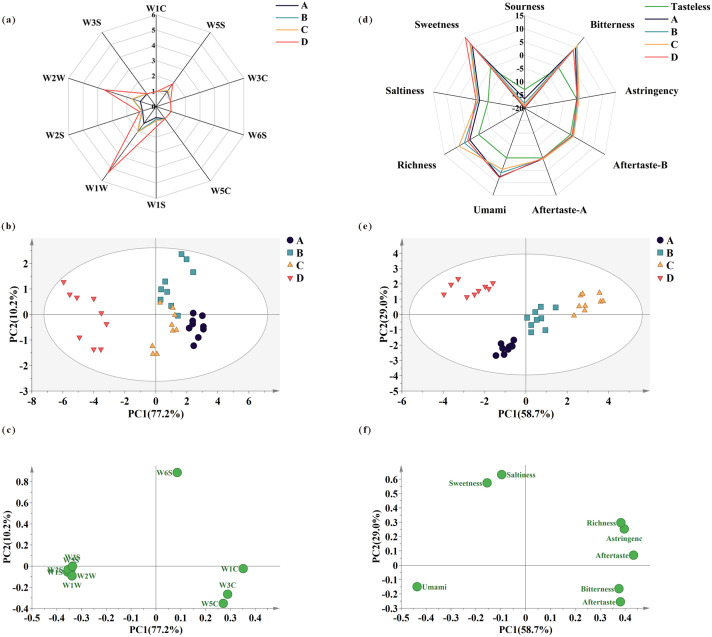
The response results of the electronic nose and electronic tongue. Radar chart of electronic nose (a) and electronic tongue (d) responses. Principal component analysis (PCA) score plot for electronic nose (b) and electronic tongue (e). PCA loading plot for electronic nose (c) and electronic tongue (f) data.

The response intensity of the electronic nose sensor reveals the flavor profile characteristics of various samples, enabling effective differentiation among them [22]. The response values are shown in a radar map (Fig 1a). Notable differences were observed among the samples, indicating that various steps influenced the flavor formation of DLSMC. The response values of group A differ from those of groups B, C, and D. The response intensity of the sensors in group B changed to varying degrees, with W1W and W2W increasing, indicating that the content of sulfur compounds has increased. Sulfur compounds contribute to the cooked flavor, primarily due to the thermal denaturation of β-lactoglobulin and milk fat globule membrane proteins, which generates thiol groups (-SH). Additionally, thermal degradation of thiamine compounds may occur when exposed to dicarbonyl compounds, including diacetyl and 2,3-pentanedione. The response intensity of W5S, W1S, W1W, W2S, and W2W exhibited an increase in group D, signifying that during the second layer of milk skin formation, VOCs including nitrogen oxides, inorganic sulfides, alcohols, aldehydes, and organic sulfur compounds were generated and amalgamated to create the characteristic flavor of DLSMC. This may be associated with changes in metabolites or the release of VOCs due to heat treatment. PCA can extract information from sensor response values, utilizing new variables to represent data features [23]. In Fig 1b, PC1 and PC2 explained 77.2% and 10.2% of the total variance, respectively, capturing the majority of the data's variability. The spatial distribution between groups A, B, and C was similar. Among them, some response values of samples in groups B and C overlapped, while there was no overlap in response values between the first three groups and group D. Group D gathers only along the negative axis of the PC1 and is associated with the sensors W5S, W1S, W1W, W2S, W2W, and W3S (Fig 1c). There was some overlap among samples A, B, and C, indicating that there was some correlation between these samples and the sensors W1C, W3C, W6S, and W5C. The analysis revealed that during the preparation of DLSMC, thermo-processing and the second-layer milk skin formation were the critical stages for flavor compound changes, while the flavor changes during the formation of the first milk skin were relatively minor.

### Electronic tongue analysis

Electronic tongue was utilized to evaluate the taste characteristics of the samples during the production process. The radar map is shown in Fig 1d. All taste indicators except sourness were valid, as the sourness of different groups was below the tasteless (−13) and was thus deemed to have no sourness [24]. The primary taste components of the samples included sweetness, bitterness, astringency, umami, richness, and saltiness, with sweetness being the most dominant across all samples. Group D exhibited comparatively elevated levels of saltiness, umami, and sweetness, while demonstrating a reduced level of bitterness compared to groups A, B, and C. As a result, the DLSMC formed after processing is saltier, fresher, sweeter, and less bitter.

Excluding sourness values, the value of other sensors was applied for PCA, and the score plot and loading plot were shown in Figs 1E and 1F. The first two principal components, PC1 and PC2, contribute 58.7% and 29.0% of the total variation. Group D, clustered in the second quadrant, is associated with sweet and savory flavors. The distances between the three groups A, B, and C were very close, indicating that the taste profiles of these three groups are relatively similar. Group A, representing raw buffalo milk, was located in the third quadrant and was associated with umami. These results indicated that the production process primarily influences bitterness, umami, richness, saltiness, and sweetness. These changes primarily occurred during the formation of the second milk skin.

### Variations in volatile flavor compounds during process of Double-Layer Steamed Milk Custard

#### Identification of volatile organic compounds via HS-GC-IMS analysis.

**Fig 2:**
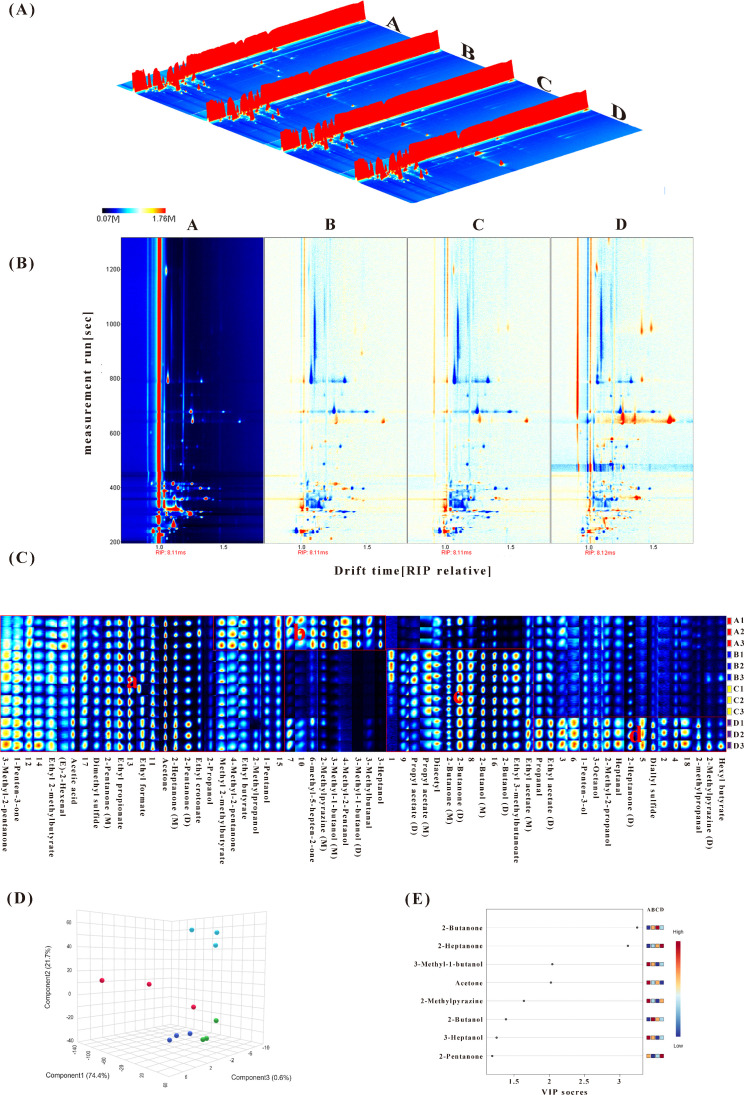
VOCs identified by HS-GC-IMS. (a) 3D topographic plot; The horizontal coordinate of the signal peak indicates the drift time of VOC, the vertical coordinate indicates measurement run time, and the volume of the signal peak indicates the concentration of VOC. (b) 2D topographic plots; The color spots on either side of the reactive ion peak (RIP) represent the response signal peaks of the substance, and their shades indicate the concentration of that substance. Using group A as a control, blue spots signify that the flavor compounds are below the values of group A, while red spots indicate higher values than group A. (c) Gallery plot. “M” represents monomer, and “D” stands for dimer. (d) PLS-DA score plots and (e) VIP values of samples during different processing.

HS-GC-IMS can obtain comprehensive ion migration spectra of samples. 64 signal peaks were detected during the process (Table 1), of which 46 were identified as known signals and 18 as unknown signals. Known compounds include aldehydes, alcohols, ketones, esters, and ethers. To understand VOC changes between samples at different stages, a 3D topographic plot was displayed (Fig 2a). Significant differences in peak positions and intensities were observed across processing stages, indicating dynamic changes in volatile profiles during preparation. The two-dimensional difference analysis (Fig 2b) revealed that the measurement run time during DLSMC production primarily occurred between 200–1,200 s. Every spot flanking the RIP peak corresponds to a VOC, with the color reflecting its concentration [25]. The signal peaks in group A were distributed between 200–800 s. Compared to group A, group B exhibited peak concentration within 200–450 s, while group C showed similar volatile signatures to group B. Group D showed the most significant changes across all stages, exhibiting more spots and darker red. This result indicates that the most significant divergence from the raw buffalo milk occurred during the second layer of milk skin formation, with a significant increase in various volatile compounds.

**Table 1:** The signal peak of VOCs detected by HS-GC-IMS.

No	Compound	CAS number	Formula	Molecular weight	Retention Index	Retention time [sec]	Drift time [a.u.]
1	1	/	/	/	748.40	212.52	1.23
2	Dimethyl sulfide	75-18-3	C_2_H_6_S	62.1	773.70	224.85	0.96
3	2-Methylpropanal	78-84-2	C_4_H_8_O	72.1	798.40	237.57	1.09
4	2	/	/	/	815.60	246.86	1.18
5	Propanal	123-38-6	C_3_H_6_O	58.1	822.20	250.51	1.14
6	Ethyl formate	109-94-4	C_3_H_6_O_2_	74.1	822.90	250.90	1.08
7	3	/	/	/	827.10	253.26	1.20
8	Acetone	67-64-1	C_3_H_6_O	58.1	854.50	269.19	1.12
9	4	/	/	/	881.50	285.91	1.20
10	2-Methyl-2-propanol	75-65-0	C_4_H_10_O	74.1	883.00	286.84	1.14
11	5	/	/	/	884.70	287.91	1.27
12	6	/	/	/	887.30	289.59	1.30
13	2-Propanol	67-63-0	C_3_H_8_O	60.1	889.00	291.42	1.22
14	Ethyl acetate (M)	141-78-6	C_4_H_8_O_2_	88.1	891.10	292.07	1.10
15	Ethyl acetate (D)	141-78-6	C_4_H_8_O_2_	88.1	891.70	292.47	1.34
16	2-Butanone (D)	78-93-3	C_4_H_8_O	72.1	908.90	303.91	1.25
17	2-Butanone (M)	78-93-3	C_4_H_8_O	72.1	909.20	304.06	1.06
18	7	/	/	/	918.60	310.49	1.33
19	8	/	/	/	918.70	310.59	1.09
20	3-Methylbutanal	590-86-3	C_5_H_10_O	86.1	920.90	312.14	1.40
21	9	/	/	/	922.30	313.11	1.22
22	10	/	/	/	922.90	313.53	1.29
23	Diacetyl	431-03-8	C_4_H_6_O_2_	86.1	927.10	316.43	1.18
24	Ethyl propionate	105-37-3	C_5_H_10_O_2_	102.1	936.40	322.11	1.14
25	11	/	/	/	958.70	339.53	1.04
26	Propyl acetate (D)	109-60-4	C_5_H_10_O_2_	102.1	977.40	354.00	1.48
27	Propyl acetate (M)	109-60-4	C_5_H_10_O_2_	102.1	977.40	354.00	1.16
28	4-Methyl-2-pentanone	108-10-1	C_6_H_12_O	100.2	984.70	356.68	1.18
29	2-Pentanone (D)	107-87-9	C_5_H_10_O	86.1	987.20	361.80	1.37
30	2-Pentanone (M)	107-87-9	C_5_H_10_O	86.1	989.30	363.47	1.12
31	Ethyl butyrate	105-54-4	C_6_H_12_O_2_	116.2	993.60	363.41	1.20
32	12	/	/	/	1002.40	375.27	1.09
33	3-Methyl-2-pentanone	565-61-7	C_6_H_12_O	100.2	1014.20	384.07	1.18
34	13	/	/	/	1014.70	387.56	1.04
35	1-Penten-3-one	1629-58-9	C_5_H_8_O	84.1	1017.30	387.42	1.10
36	Methyl 2-methylbutyrate	868-57-5	C_6_H_12_O_2_	116.2	1023.40	393.94	1.19
37	14	/	/	/	1023.80	397.00	1.39
38	Ethyl 2-methylbutyrate	7452-79-1	C_7_H_14_O_2_	130.2	1024.60	395.30	1.23
39	2-Butanol (D)	78-92-2	C_4_H_10_O	74.1	1026.90	397.81	1.32
40	2-Butanol (M)	78-92-2	C_4_H_10_O	74.1	1027.50	398.42	1.15
41	15	/	/	/	1032.30	406.03	1.04
42	16	/	/	/	1041.00	415.38	1.11
43	Ethyl 3-methylbutanoate	108-64-5	C_7_H_14_O_2_	130.2	1041.00	413.50	1.26
44	2-Methyl-1-propanol	78-83-1	Formula	74.1	1099.70	486.81	1.17
45	Diallyl sulfide	592-88-1	/	114.2	1136.40	546.49	1.13
46	17	/	C_2_H_6_S	/	1136.60	546.32	1.07
47	Ethyl crotonate	623-70-1	C_4_H_8_O	114.1	1151.90	573.77	1.18
48	1-Penten-3-ol	616-25-1	/	86.1	1168.60	604.72	0.94
49	18	/	C_3_H_6_O	/	1188.30	644.39	1.66
50	2-Heptanone (D)	110-43-0	C_3_H_6_O_2_	114.2	1188.90	644.79	1.63
51	2-Heptanone (M)	110-43-0	/	114.2	1189.10	645.19	1.26
52	Heptanal	111-71-7	C_3_H_6_O	114.2	1190.30	647.67	1.34
53	4-Methyl-2-pentanol	108-11-2	/	102.2	1211.10	675.85	1.29
54	3-Methyl-1-butanol (M)	123-51-3	C_4_H_10_O	88.1	1213.90	679.50	1.25
55	3-Methyl-1-butanol (D)	123-51-3	/	88.1	1214.90	680.89	1.50
56	(E)-2-Hexenal	6728-26-3	/	98.1	1257.00	739.19	1.18
57	1-Pentanol	71-41-0	C_3_H_8_O	88.1	1259.10	742.15	1.26
58	3-Heptanol	589-82-2	C_4_H_8_O_2_	116.2	1291.50	790.62	1.33
59	2-Methylpyrazine (D)	109-08-0	C_4_H_8_O_2_	94.1	1292.20	791.79	1.41
60	2-Methylpyrazine (M)	109-08-0	C_4_H_8_O	94.1	1292.20	791.79	1.07
61	6-Methyl-5-hepten-2-one	110-93-0	C_4_H_8_O	126.2	1293.80	794.28	1.17
62	3-Octanol	589-98-0	/	130.2	1390.90	967.09	1.40
63	Hexyl butyrate	2639-63-6	/	172.3	1395.40	976.01	1.49
64	Acetic acid	64-19-7	C_5_H_10_O	60.1	1494.20	1192.46	1.06

**Note**: The signal peaks of VOCs after headspace extraction were detected successively due to their different mobility, and some single compounds may produce multiple signals of dimers depending on their concentration. (M) monomer, (D) dimer.

#### Volatile organic compounds in different processing stages of Double-Layer Steamed Milk Custard.

All peaks were selected to compare VOCs across different samples. The Gallery Plot (Fig 2c) displays comprehensive VOC information and illustrates the dynamic changes in VOCs. Esters and alcohols were the most abundant VOCs detected during the production of DLSMC, followed by ketones and aldehydes. 25 signal peaks were detected across all 4 groups, with 18 VOCs identified, including 6 ketones, 6 esters, 3 alcohols, 1 ether, 1 acid, and 1 aldehyde, corresponding to region (Ⅰ) of the plot. Ketones are typical VOCs in various types of milk, with acetone being an important carbonyl compound that imparts a slightly sweet fruity flavor. 2-Pentanone and 2-heptanone are characterized by sweet and creamy flavors, respectively. Ketones primarily derive from the β-oxidation series reactions of saturated fatty acids in milk fat [26]. Esters are common VOCs in milk and dairy products, potentially arising from the esterification reaction of fatty acids and free alcohols released by hydrolysis in the lactation system under the action of endogenous esterase. Aldehydes mainly originate from lipid oxidation in buffalo milk [27].

Methyl 2-methylbutyrate, 4-methyl-2-pentanone, ethyl butyrate, 2-methylpropanol, 1-pentanol, 6-methyl-5-hepten-2-one, 2-methylpyrazine (M), 3-methyl-1-butanol (M), 4-methyl-2-pentanol, 3-methyl-1-butanol (D), 3-methylbutanal, and 3-heptanol are present in raw buffalo milk, with their contents decreasing after thermo-processing, marked as region (Ⅱ). 3-Heptanol and 4-methyl-2-pentanol are only found in raw buffalo milk, indicating that these substances may be the characteristic VOCs of raw buffalo milk. Region (Ⅲ) represents flavor compounds produced or significantly increased during thermo-processing. A minimal quantity of diacetyl was detected in raw buffalo milk (group A), whereas an increase in diacetyl content was observed in group B. Previous research has indicated that diacetyl exists in raw buffalo milk at 65.2 mg/kg [28]. And diacetyl is a compound characterized by its butter-like flavor, which has been demonstrated to form in milk as a result of heat treatment [29]. Diacetyl, acetone, and 2-butanone may originate from lipid oxidation or the degradation of reducing sugars into carbonyl compounds [30].

In DLSMC, 60 peaks were detected. After the second-layer milk skin formation, the concentrations of propanal, ethyl acetate (D), 1-penten-3-ol, 3-octanol, 2-methyl-2-propanol, heptanal, 2-heptanone (D), diallyl sulfide, 2-methyl-1-propanol, 2-methylpyrazine (D), and hexyl butyrate increased, corresponding to region (Ⅳ). Ketones are mainly produced by fat degradation during thermal processing and decarboxylation reactions after the oxidation of free fatty acids to β-ketoacids. These compounds are important flavor contributors in dairy products [13,26]. 2-Heptanone, produced from β-keto acids, is the most common compound in the heat treatment of milk. Compared to raw milk, ultrahigh-temperature sterilization also significantly increased the levels of methyl ketones, including 2-heptanone, leading to the formation of oxidized flavors in the milk [31]. Aldehydes, which may result from milk fat oxidation, contribute to milk flavor due to their strong volatility, high concentration, and low threshold. Heptanal is primarily formed by the oxidation of linoleic and arachidonic acids, while octanal and nonanal are primarily formed by the oxidation of oleic acid. Most of these compounds may result from the Maillard reaction or the thermal degradation of fat during making. Leucine and isoleucine produce 3-methylbutyral and 2-methylbutyral, respectively, through Strecker degradation, providing a fatty aroma and nutty taste [32]. Ester compounds, especially fatty acid ethyl esters, exhibit fruity and sweet flavors that can reduce the bitterness brought by fatty acids and amines [33]. As shown in the electronic tongue results, the bitterness of group D was the lowest, possibly resulting from a rise in the variety and concentration of ester compounds. This increase reduced the bitterness, enriched the flavor profile of DLSMC, and optimized its sensory quality.

#### The key flavor compounds during different processing.

The PLS-DA model was established (Fig 2d), and the combined variance explained by the top three PCs was 96.7%, providing a strong basis for the model's discriminative ability. The 3D score map illustrated distinctions among the four groups, indicating differences in the types and contents of aroma compounds across them. The VIP value can further evaluate and classify the parameters, distinguishing different groups. In total, 8 compounds with VIP > 1 were identified as key VOCs for distinguishing aroma compounds among the groups (Fig 2e). VOCs such as 2-butanone, 2-heptanone, 3-methyl-1-butanol, acetone, 2-methylpyrazine, 2-butanol, 2-pentanone, and 3-heptanol significantly impacted the flavor characteristics of DLSMC. Ketones, which account for 62.50% of all key flavor compounds, are considered the primary contributors to the creamy and sweet taste in dairy products [13]. 2-Butanone has been identified as the compound responsible for the egg flavor. This oxygenated compound is primarily formed as a result of lipid oxidation [34]. After thermal treatment, the content of 2-butanone in the sample changed the most, making it a key compound for the study of DLSMC made from buffalo milk. Ketones are mainly derived from the β-oxidation reactions of saturated fatty acids in milk fat. This suggests that the change in flavor substances in DLSMC may be associated with fatty acid oxidation.

### Changes in metabolites during the processing of Double-Layer Steamed Milk Custard

#### Analysis of metabolite differences and pathways during processing of Double-Layer Steamed Milk Custard.

Raw data undergoes pre-processing steps such as identification, filtering, complementing, normalization, and logarithmic transformation, which can eliminate or reduce errors caused by experiments and analysis. The VIP value can be used to identify metabolites that significantly contribute to differences between groups. According to OPLS-DA, criteria of VIP > 1 and p < 0.05 were used to search for metabolites that were significantly differentially expressed between groups A and D. Overall, 913 distinct metabolites were identified, including amino acids, nucleotides, lipids, and other metabolites. For additional details, please consult the supplementary material (S3 Fig). We investigated buffalo milk metabolic pathways during processing, potentially involving amino acid metabolism, lipid metabolism, and glycolysis/gluconeogenesis.

**Fig 3:**
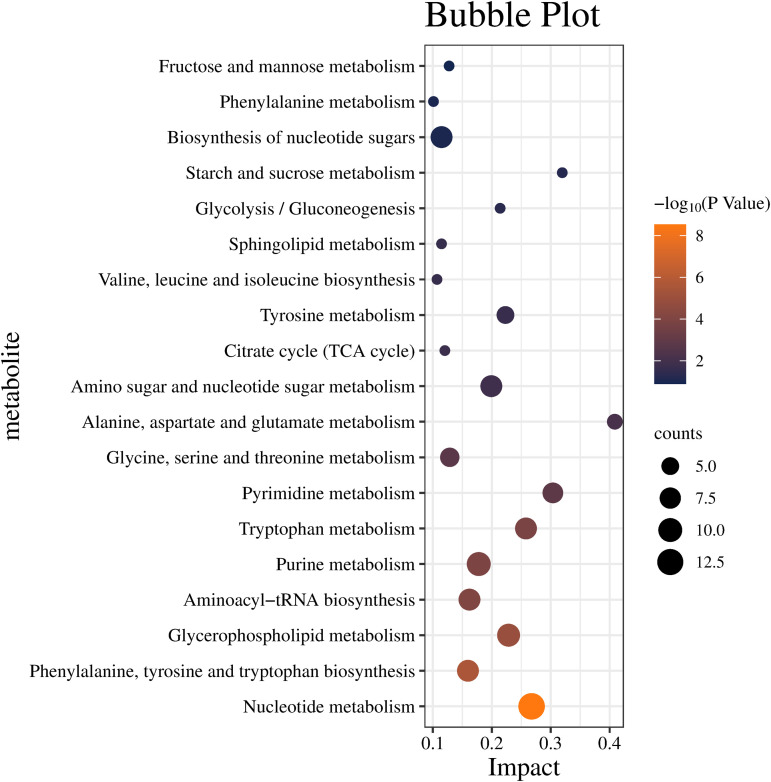
Pathway enrichment analysis of differential metabolites of Double-Layer Steamed Milk Custard during different processing.

For a deeper analysis of the potential metabolic pathways involved in the flavor formation of DLSMC production, KEGG database was used to analyze the functions of pathways related to differential metabolites. As shown in the S2 Table, a total of 913 different metabolites were enriched in 60 metabolic pathways. There were 19 significantly enriched metabolic pathways with an impact value greater than 0.1 (Fig 3). The results showed that the effects of nucleotide metabolism were the most significant. Additionally, metabolic pathways such as phenylalanine, tyrosine and tryptophan biosynthesis, glycerophospholipid metabolism, purine metabolism, tryptophan metabolism, pyrimidine metabolism, glycine, serine and threonine metabolism were also significantly enriched. These pathways involve nucleotides, amino acids, and lipids, suggesting that these compounds may primarily contribute to the significant differences in VOCs from buffalo milk to DLSMC.

Colors (varying from blue to orange) denote increased significance levels, and the horizontal coordinate indicates the impact value of the pathway.

#### Analysis of metabolite differences during the processing of Double-Layer Steamed Milk Custard.

**Fig 4:**
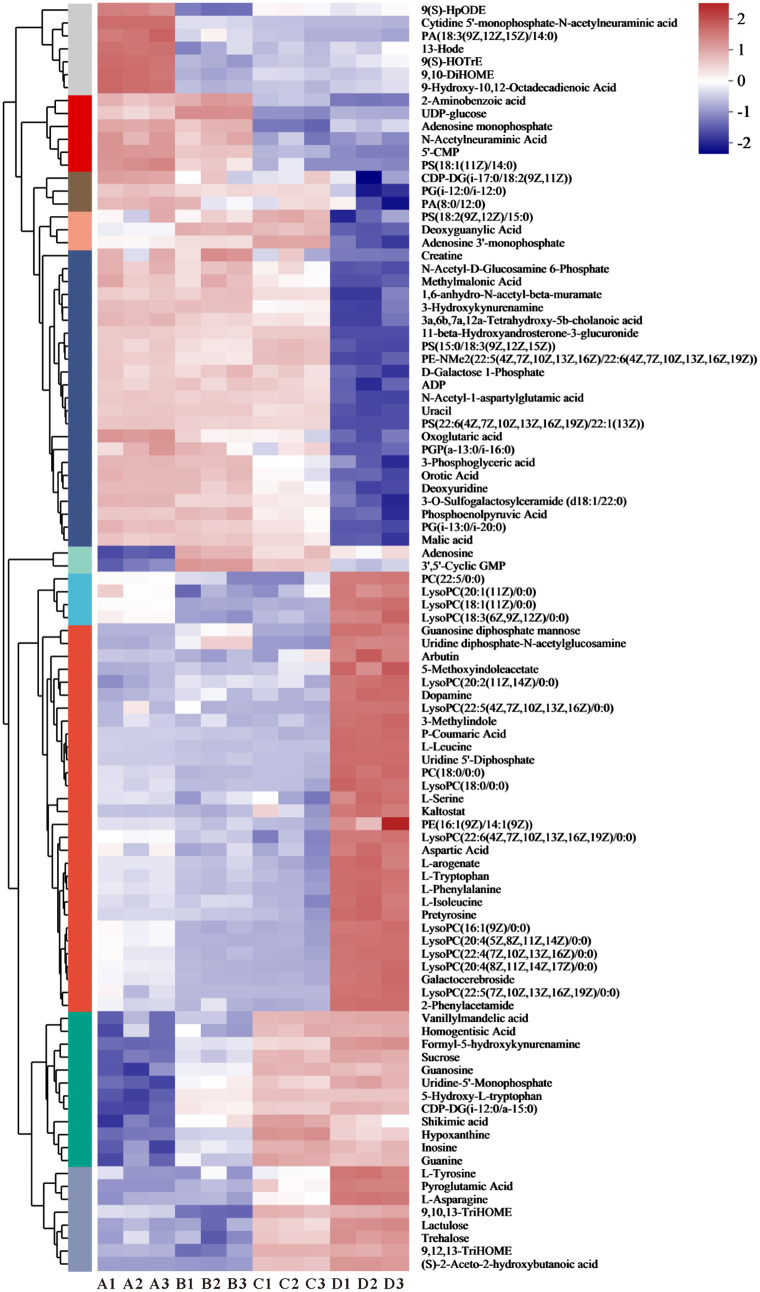
Changes in metabolites during the processing of Double-Layer Steamed Milk Custard.

To better visualize sample relationships and variations in metabolite expression, the relative values of metabolites across different groups were used as metabolic levels, and 98 metabolites enriched in pathways were analyzed using hierarchical clustering. Data differences are represented using color gradients (Fig 4.).

Milk is a complex food matrix that is prone to lipolysis and oxidation under heat treatment [35]. Linoleic acid derived 9,10-DiHOME, 13-HODE, 9(S)-HpODE, and α-linolenic acid derived 9(S)-HOTrE initially decreased during the production process before gradually increasing and stabilizing, while linoleic acid derived 9,10,13-TriHOME and 9,12,13-TriHOME were highly expressed in groups C and D. HpODE is a chemically unstable primary oxidation product of linoleic acid, while stable secondary oxidation products, such as HODE and TriHOME can be generated through non-enzymatic reduction [36]. Dietary HODEs can be directly absorbed, leading to increased levels of HODEs in the body, but the extent to which exogenous HODEs contribute to the physiological effects of HODEs is unclear [37]. Stirring and heat treatment may cause partial disruption of the milk fat globule membrane (MFGM), releasing components such as triglycerides, phospholipids, and proteins [38]. The protein and phospholipid components released after MFGM rupture are more easily digested and absorbed by the human body [39].

Phosphatidylserine (PS), phosphatidylethanolamine (PE), phosphatidylglycerol (PG), and phosphatidic acid (PA) were observed to gradually decrease in Fig 4. With the heat treatment, the oxidization of milk lipids was aggravated and more oxidized fatty acids were released [40]. DLSMC contains a rich variety and high levels of lysophosphatidylcholine (LPC) compounds. Some may have been formed by oxidation during heat treatment, while others may have resulted from the addition of eggs. Eggs also contain lysophospholipids, namely lysophosphatidylcholine (LPC) and lysophosphatidylethanolamine (LPE) [34]. Docosahexaenoic acid (DHA) is highly concentrated in the brain and its deficiency is associated with several neurological disorders including Alzheimer's disease. The enrichment of docosahexaenoic acid (DHA) in the brain is highly dependent on the molecular carrier of dietary DHA, with studies showing that LPC is more effective than phosphatidylcholine or triacylglycerol [41].

Free amino acids can be absorbed and utilized by the human body, and their content and composition can partly reflect the nutritional value of food. According to the results, most free amino acids were present at higher levels in DLSMC. Aspartic acid, an MSG-like component, imparted an umami flavor to DLSMC, while serine and alanine contributed a pleasant sweet taste [42]. This may be one of the reasons why the DLSMC is sweeter. Aromatic amino acids such as tryptophan, phenylalanine, and tyrosine. Shikimic acid, an important intermediate in the biosynthesis of aromatic amino acids, gradually increases in content during the production process. Tryptophan is an essential amino acid that must be ingested through food and is a precursor to the synthesis of various biologically important compounds. In the body, tryptophan is converted to 5-hydroxytryptophan (5-HTP) and serotonin (5-HT), playing a crucial role in regulating adaptive responses to environmental changes, including sleep, cognition, and feeding behavior [43]. Cysteine in whey protein is a precursor to glutathione, an important antioxidant in the body, and taurine, which can clear free radicals in the human body [44]. Pyroglutamate, a metabolite of the glutathione cycle, is highly expressed in DLSMC. It can participate in energy metabolism, provide energy, and has antioxidant, anti-inflammatory, and other immune functions.

In addition to the aforementioned metabolites, we also identified various nucleotides involved in nucleotide metabolism among the differential metabolites. Exogenous nucleotides (NTs) were essential nutrients under specific physiological conditions, including immune responses, hunger, rapid growth, and aging. Exogenous NTs can be absorbed by human tissues, thereby reducing the body's need for de novo synthesis or salvage synthesis [45]. 5'-Cytidine monophosphate (5’-CMP) is a flavor nucleotide that, as a single product, does not have umami. However, when mixed with sodium glutamate, its umami is much greater than the sum of the umami of the individual products [46]. Although the content of 5’-CMP gradually decreased and the umami taste slightly diminished during the production process, the umami taste of DLSMC was enhanced by the addition of free amino acids. The combined action of various umami substances made DLSMC rich in flavor. Guanosine and inosine are two nucleoside compounds that gradually increase in expression during processing. Guanosine is formed by the combination of guanine and ribose and can be phosphorylated to become guanosine monophosphate (GMP), cyclic guanosine monophosphate (cGMP), guanosine diphosphate (GDP), or guanosine triphosphate (GTP). These compounds have various physiological functions in the body, including participating in RNA synthesis and metabolism, providing energy, and affecting the immune system. Inosine, formed by combining hypoxanthine with ribose, also has various physiological functions in the human body, including participating in RNA synthesis and metabolism, providing energy, and affecting the immune system.

The addition of sucrose significantly enhanced the sweetness of DLSMC. Glucose, trehalose, and lactulose also contributed to its sweetness. UDP-glucose exhibited a gradual decrease while trehalose demonstrated a gradual increase. This is because UDP-glucose may generate alginose through Starch and sucrose metabolism under conditions such as temperature. Furthermore, lactulose exhibited high expression after heat treatment. Lactulose is an isomer of lactose, and its concentration can be used as an indicator of heat damage to describe milk heat treatment. Lactulose is not metabolized by oral bacteria and is therefore not cariogenic. Additionally, it can regulate the intestinal microbial community and prevent the growth of harmful spoilage bacteria [47]. 3-phosphoglyceric acid is a link in the glycolysis process, containing a high-energy phosphate bond, and its expression gradually decreases during processing. Moreover, 3-phosphoglyceric acid is an intermediate in serine synthesis. Buffalo milk, rich in protein and sugar, undergoes the Maillard reaction during heat treatment, which leads to the production of various VOCs.

### Relevance analysis of key volatile organic compounds and differential metabolites

**Fig 5:**
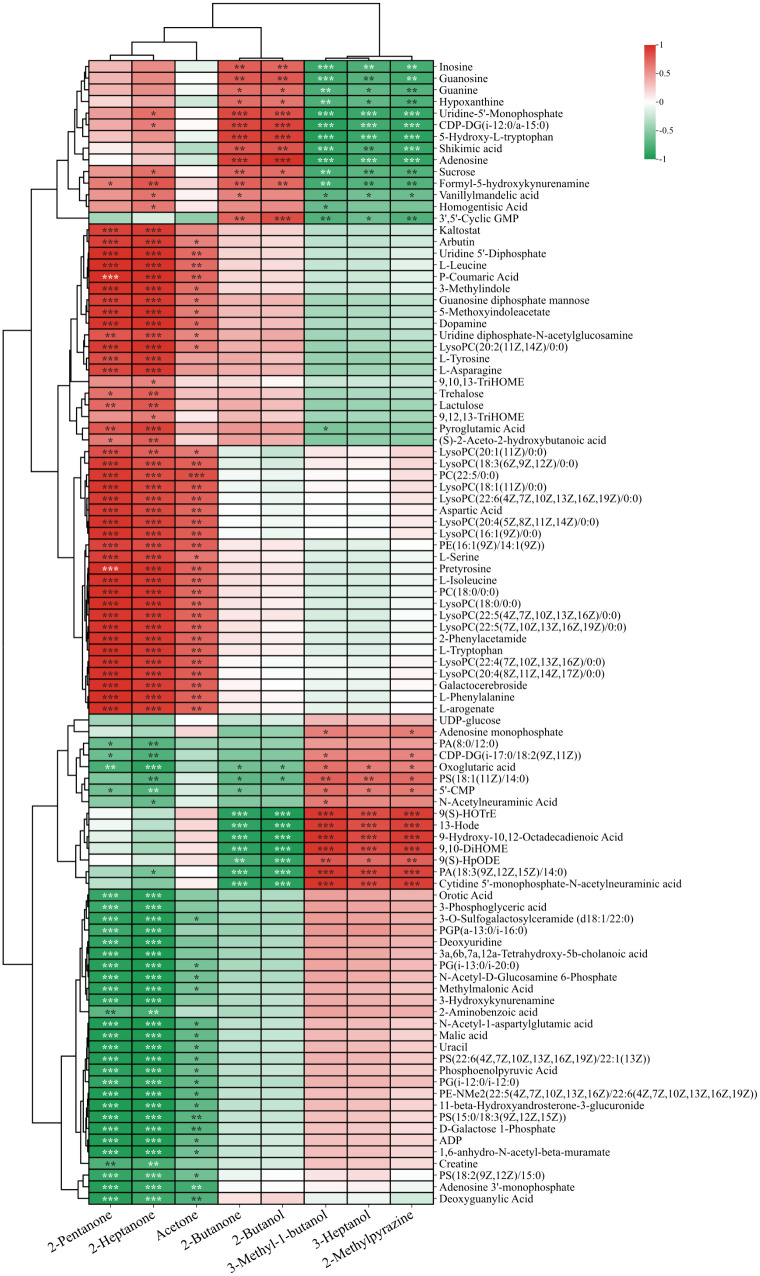
Relationship between key volatile organic compounds and differential metabolites.

The formation of VOCs during processing is a complicated process, and differential metabolites may be potential factors affecting the flavor of DLSMC. The correlation between 8 key VOCs with VIP > 1 and 98 different metabolites is shown in Fig 5. The results showed that LPC has strong positive correlation with 2-pentanone, 2-heptanone and acetone, while PG, PS, PA showed negatively correlation with 2-pentanone, 2-heptanone and acetone. The oxidation product of linoleic acid and α-linolenic acid were negative correlation with 2-butanone and 2-butanol and have positive correlation with 3-methyl-1-butanol, 3-heptanol and 2-methylpyrazine. Many aromas typically found in milk are generated from short- and medium-chain fatty acids present in milk fat. Unsaturated fatty acids are transformed into aldehydes, acids, and alcohols, whereas the free fatty acids are transformed into esters [48]. Amino acids also had a significant influence on the flavors. The most amino acids were positively correlated with 2-pentanone, 2-heptanone, acetone. This may result from the production of VOCs, such as ketones, in a complex series of non-enzymatic browning reactions between reducing sugars and free amino acids during the Maillard reaction [49]. Glycogen was also an important volatile flavor precursor substance. Sucrose and glucose were negatively correlated with 2-methylpyrazine. Pyrazines, important products of the Maillard reaction, impart a sweet and egg-like aroma to DLSMC [34].

In summary, the results indicate that amino acids, lipids, and glycogen, as precursors, significantly influence the VOCs of DLSMC. Throughout the DLSMC production process, not all metabolites were associated with VOCs, and some showed limited or no involvement in the formation of key volatile compounds. Generally, the reason for the flavor formation during production of buffalo milk Double-Layer Steamed Milk Custard was the combination of physicochemical actions such as oxidative decomposition of lipids and proteins, and Maillard reaction under high temperature. The unique flavor of DLSMC results from complex interactions among a wide variety of compounds originating from numerous chemical and biochemical reactions involving proteins, fats, and carbohydrates.

## Conclusion

This study comprehensively investigated the flavor formation and metabolic changes during the production of DLSMC. The results revealed that thermo-processing and the formation of the second milk skin layer are critical stages for flavor formation. Sulfur compounds, ketones, esters, and aldehydes were the main VOCs during the processing of DLSMC. HS-GC-IMS identified 46 VOCs, and selected 8 key VOCs (VIP > 1): 2-butanone, 2-heptanone, 3-methyl-1-butanol, acetone, 2-methylpyrazine, 2-butanol, 2-pentanone, and 3-heptanol. Metabolomics analysis revealed differentially expressed metabolites, which were enriched in pathways related to nucleotide metabolism, amino acid metabolism, and lipid metabolism, significantly contributing to changes in key compounds. The Maillard reaction, lipid oxidation, and protein degradation under heat treatment were identified as the primary drivers of flavor changes. These findings provide insights into the flavor formation mechanisms and highlight the importance of specific processing stages in optimizing the sensory quality of DLSMC. However, the characteristic flavor compounds in DLSMC and the specific mechanisms underlying their changes require further study.

## Supporting information

S1 Fig HS-GC-IMS 2D topographic plots of the ketone mixture (C4-C9). (TIF)

S2 Fig Calibration curve of the ketone mixture (C4-C9). (TIF)

S3 Fig Differential metabolites between groups A and D. (TIF)

S1 Table Sensor properties of electronic nose. (DOCX)

S2 Table KEGG topology analysis. (DOCX)

S1 Dataset Electronic nose, electronic tongue, and HS-GC-IMS. (XLSX)

S2 Dataset Non-targeted metabolomics data. (DOCX)

## Abbreviations

DLSMC: Double-layer steamed milk custard
VOCs: Volatile organic compounds
HS-GC-IMS: Headspace-gas chromatography-ion mobility spectrometry
PCA: Principal component analysis
OPLS-DA: Partial least squares-discriminant analysis
VIP: Variable importance in projection
