# Supplementary figures and images for: Analysis of flavor formation and metabolite changes during production of Double-Layer Steamed Milk Custard made from buffalo milk
Source: PLoS One. 2025 Sep 8;20(9):e0331277. doi: 10.1371/journal.pone.0331277 (PMC12416662; doi:10.1371/journal.pone.0331277)

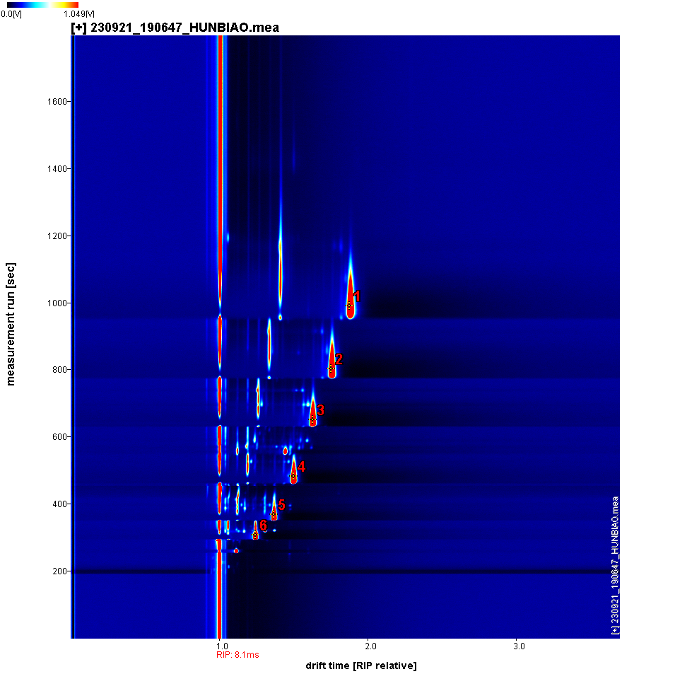

Supplement: S1 Fig — (TIF) [file pone.0331277.s001.tif]

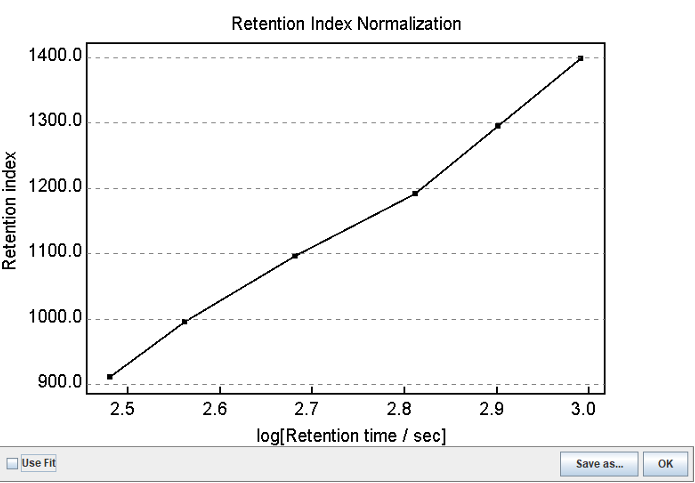

Supplement: S2 Fig — (TIF) [file pone.0331277.s002.tif]

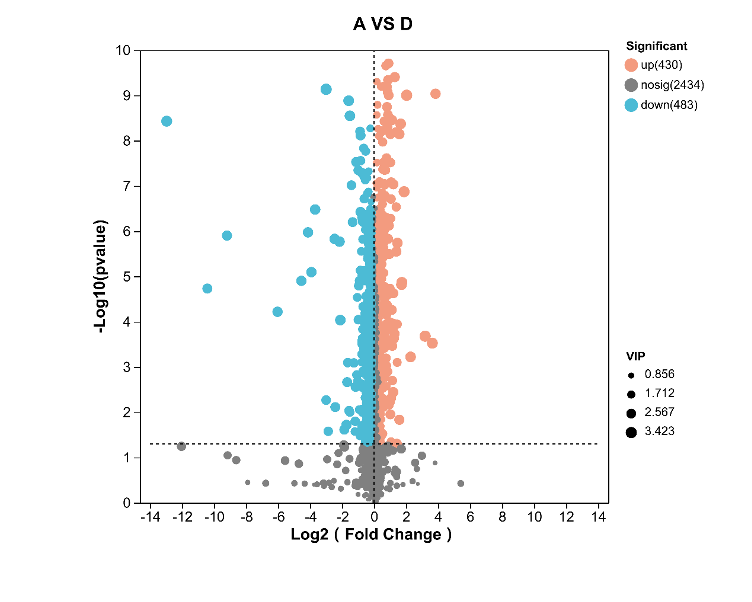

Supplement: S3 Fig — (TIF) [file pone.0331277.s003.tif]
