# Supplementary material for: Analysis of flavor formation and metabolite changes during production of Double-Layer Steamed Milk Custard made from buffalo milk
Source: PLoS One. 2025 Sep 8;20(9):e0331277. doi: 10.1371/journal.pone.0331277 (PMC12416662; doi:10.1371/journal.pone.0331277)
Supplement: S1 Table — (DOCX) [file pone.0331277.s004.docx]

Table S1. Sensor properties of electronic nose

| Sensor number in array | Sensor | General description |
| --- | --- | --- |
| S1 | W1C | nitrogen oxides |
| S2 | W5S | methyl |
| S3 | W3C | alcohols, ketones and aldehydes |
| S4 | W6S | ammonia |
| S5 | W5C | benzene |
| S6 | W1S | short-chain aromatic compounds and olefin |
| S7 | WIW | sulfur compounds |
| S8 | W2S | organic sulfides |
| S9 | W2W | hydrogen |
| S10 | W3S | long-chain alkanes |
