# Supplementary material for: Analysis of flavor formation and metabolite changes during production of Double-Layer Steamed Milk Custard made from buffalo milk
Source: PLoS One. 2025 Sep 8;20(9):e0331277. doi: 10.1371/journal.pone.0331277 (PMC12416662; doi:10.1371/journal.pone.0331277)
Supplement: S2 Table — (DOCX) [file pone.0331277.s005.docx]

Table S2 KEGG topology analysis

| No | Pathway_ID | Pathway Description | Impact_value | P_value | P_adjust |
| --- | --- | --- | --- | --- | --- |
| 1 | map00250html | Alanine, aspartate and glutamate metabolism | 0.408695652 | 0.006710174 | 0.036600947 |
| 2 | map00500html | Starch and sucrose metabolism | 0.319570851 | 0.060534713 | 0.134521585 |
| 3 | map00240html | Pyrimidine metabolism | 0.303633615 | 0.001455895 | 0.010919216 |
| 4 | map01232html | Nucleotide metabolism | 0.267562537 | 2.97E-09 | 8.91E-08 |
| 5 | map00380html | Tryptophan metabolism | 0.258043607 | 0.000140456 | 0.00120391 |
| 6 | map00564html | Glycerophospholipid metabolism | 0.228495407 | 1.14E-05 | 0.000170625 |
| 7 | map00350html | Tyrosine metabolism | 0.223198474 | 0.020857619 | 0.073615127 |
| 8 | map00010html | Glycolysis / Gluconeogenesis | 0.214215686 | 0.046617576 | 0.11654394 |
| 9 | map00520html | Amino sugar and nucleotide sugar metabolism | 0.199229237 | 0.012347288 | 0.052916949 |
| 10 | map00230html | Purine metabolism | 0.177890581 | 0.000120281 | 0.001202805 |
| 11 | map00970html | Aminoacyl-tRNA biosynthesis | 0.162162162 | 8.31E-05 | 0.000997254 |
| 12 | map00400html | Phenylalanine, tyrosine and tryptophan biosynthesis | 0.159566375 | 2.59E-06 | 5.18E-05 |
| 13 | map00260html | Glycine, serine and threonine metabolism | 0.128734827 | 0.001698057 | 0.011320383 |
| 14 | map00051html | Fructose and mannose metabolism | 0.127613837 | 0.125505111 | 0.209175185 |
| 15 | map00020html | Citrate cycle (TCA cycle) | 0.12041944 | 0.016189814 | 0.064759257 |
| 16 | map01250html | Biosynthesis of nucleotide sugars | 0.114771271 | 0.090664658 | 0.181329315 |
| 17 | map00600html | Sphingolipid metabolism | 0.114754098 | 0.023086423 | 0.074874885 |
| 18 | map00290html | Valine, leucine and isoleucine biosynthesis | 0.107058824 | 0.023086423 | 0.074874885 |
| 19 | map00360html | Phenylalanine metabolism | 0.101131241 | 0.090753269 | 0.175651487 |
| 20 | map00770html | Pantothenate and CoA biosynthesis | 0.099919743 | 0.034011589 | 0.092758878 |
| 21 | map00780html | Biotin metabolism | 0.097799511 | 0.119717123 | 0.205229353 |
| 22 | map00620html | Pyruvate metabolism | 0.091409861 | 0.146116914 | 0.219175371 |
| 23 | map00565html | Ether lipid metabolism | 0.081504702 | 0.33755743 | 0.413335628 |
| 24 | map00100html | Steroid biosynthesis | 0.06809167 | 0.140315098 | 0.21867288 |
| 25 | map00310html | Lysine degradation | 0.066634708 | 0.032061871 | 0.091605346 |
| 26 | map00270html | Cysteine and methionine metabolism | 0.063775869 | 0.061717369 | 0.132251506 |
| 27 | map00052html | Galactose metabolism | 0.063055206 | 0.007779942 | 0.038899711 |
| 28 | map00130html | Ubiquinone and other terpenoid-quinone biosynthesis | 0.060999172 | 0.170618153 | 0.249685102 |
| 29 | map00524html | Neomycin, kanamycin and gentamicin biosynthesis | 0.055439458 | 0.106956241 | 0.194465893 |
| 30 | map00630html | Glyoxylate and dicarboxylate metabolism | 0.055042735 | 0.049465068 | 0.118716162 |
| 31 | map00470html | D-Amino acid metabolism | 0.053892216 | 0.054260991 | 0.125217672 |
| 32 | map00140html | Steroid hormone biosynthesis | 0.047539093 | 0.028151973 | 0.08445592 |
| 33 | map00561html | Glycerolipid metabolism | 0.04683013 | 0.010520301 | 0.048555237 |
| 34 | map00330html | Arginine and proline metabolism | 0.041661975 | 0.276428997 | 0.360559561 |
| 35 | map00740html | Riboflavin metabolism | 0.035799523 | 0.306851492 | 0.383564365 |
| 36 | map01240html | Biosynthesis of cofactors | 0.034037697 | 0.003256413 | 0.019538476 |
| 37 | map00280html | Valine, leucine and isoleucine degradation | 0.023862876 | 0.079183229 | 0.163827371 |
| 38 | map00040html | Pentose and glucuronate interconversions | 0.014290407 | 0.140315098 | 0.21867288 |
| 39 | map00430html | Taurine and hypotaurine metabolism | 0.010342084 | 0.106135775 | 0.199004578 |
| 40 | map00120html | Primary bile acid biosynthesis | 0.008193288 | 0.357420222 | 0.420494379 |
| 41 | map00480html | Glutathione metabolism | 0.001802745 | 0.372753648 | 0.385607222 |
| 42 | map00220html | Arginine biosynthesis | 0 | 0.112945884 | 0.199316265 |
| 43 | map00590html | Arachidonic acid metabolism | 0 | 0.37366249 | 0.379995752 |
| 44 | map00730html | Thiamine metabolism | 0 | 0.369564802 | 0.403161602 |
| 45 | map00071html | Fatty acid degradation | 0 | 0.354694413 | 0.425633295 |
| 46 | map00030html | Pentose phosphate pathway | 0 | 0.374641903 | 0.368500232 |
| 47 | map00591html | Linoleic acid metabolism | 0 | 0.263468735 | 0.351291647 |
| 48 | map00440html | Phosphonate and phosphinate metabolism | 0 | 0.366692137 | 0.407435708 |
| 49 | map00340html | Histidine metabolism | 0 | 0.374387683 | 0.374387683 |
| 50 | map00760html | Nicotinate and nicotinamide metabolism | 0 | 0.250805718 | 0.349961467 |
| 51 | map00982html | Drug metabolism - cytochrome P450 | 0 | 0.263032987 | 0.358681345 |
| 52 | map00650html | Butanoate metabolism | 0 | 0.362405773 | 0.418160507 |
| 53 | map00900html | Terpenoid backbone biosynthesis | 0 | 0.364642455 | 0.41280278 |
| 54 | map00510html | N-Glycan biosynthesis | 0 | 0.019236093 | 0.072135349 |
| 55 | map00053html | Ascorbate and aldarate metabolism | 0 | 0.042578893 | 0.111075372 |
| 56 | map00410html | beta-Alanine metabolism | 0 | 0.126433737 | 0.205027682 |
| 57 | map00920html | Sulfur metabolism | 0 | 0.371361442 | 0.39788726 |
| 58 | map00983html | Drug metabolism - other enzymes | 0 | 0.371587101 | 0.391144316 |
| 59 | map00670html | One carbon pool by folate | 0 | 0.204781261 | 0.292544658 |
| 60 | map00980html | Metabolism of xenobiotics by cytochrome P450 | 0 | 0.276645013 | 0.353163847 |
