# Supplementary material for: Analysis of flavor formation and metabolite changes during production of Double-Layer Steamed Milk Custard made from buffalo milk
Source: PLoS One. 2025 Sep 8;20(9):e0331277. doi: 10.1371/journal.pone.0331277 (PMC12416662; doi:10.1371/journal.pone.0331277)
Supplement: S2 Dataset — (DOCX) [file pone.0331277.s007.docx]

Metabolomic raw data were uploaded ( ID: MTBLS12717) ( MetaboLights：<https://www.ebi.ac.uk/metabolights/>).

Please refer to <https://www.ebi.ac.uk/metabolights/reviewerce228ac0-a196-4121-95fb-d8f6545af614>.
